# Fitz Hugh Curtis Case Report

**DOI:** 10.21980/J82K9G

**Published:** 2020-04-15

**Authors:** Savannah Loehr, Cindy Bitter

**Affiliations:** * Saint Louis University School of Medicine, St Louis, MO; * SSM Saint Louis University Hospital, Department of Emergency Medicine, St Louis, MO

## Abstract

Fitz-Hugh Curtis syndrome (FHCS) is a rare sequela of pelvic inflammatory disease that must be included on the differential in patients with abdominal pain, particularly if they have risk factors for sexually transmitted infections. In this case, a 25-year-old female with a past history of *Chlamydia* presented to the emergency department with vaginal discharge and right upper quadrant pain. Complete blood count showed a mild leukocytosis, and computed tomography demonstrated fat stranding inferior to the liver and along the right colon. The patient was diagnosed with Fitz-Hugh Curtis syndrome and admitted for intravenous (IV) antibiotics. After 48 hours of IV antibiotics she was discharged with a 14-day course of doxycycline and metronidazole. Multiple bacterial species have been implicated in FHCS, including but not limited to *Chlamydia trachomatis*, *Neisseria gonorrheae*, *Mycoplasma genitalium*, *Peptostreptococcus spp.*, and *Prevotella spp*. Therefore, careful consideration should be given to the choice of antimicrobial treatment.

**Topics:**

Fitz-Hugh-Curtis syndrome, perihepatitis, pelvic inflammatory disease, sexually transmitted disease.

[Fig f1-jetem-5-2-v19]

**Figure f1-jetem-5-2-v19:**
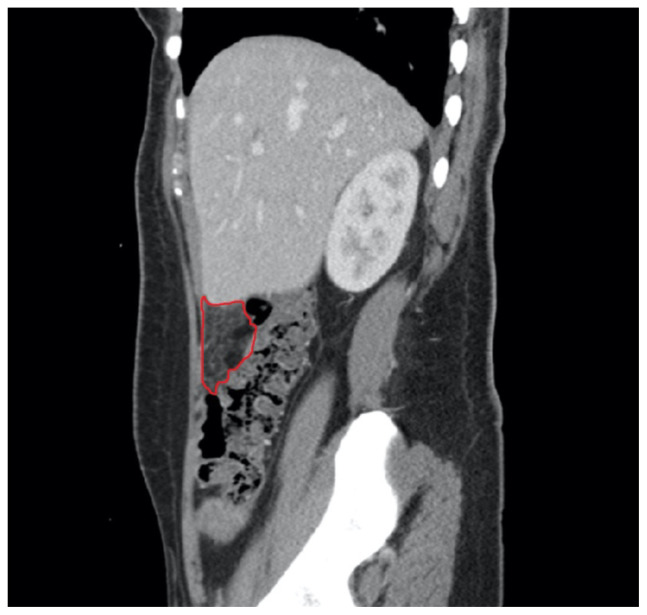

[Fig f2-jetem-5-2-v19]

**Figure f2-jetem-5-2-v19:**
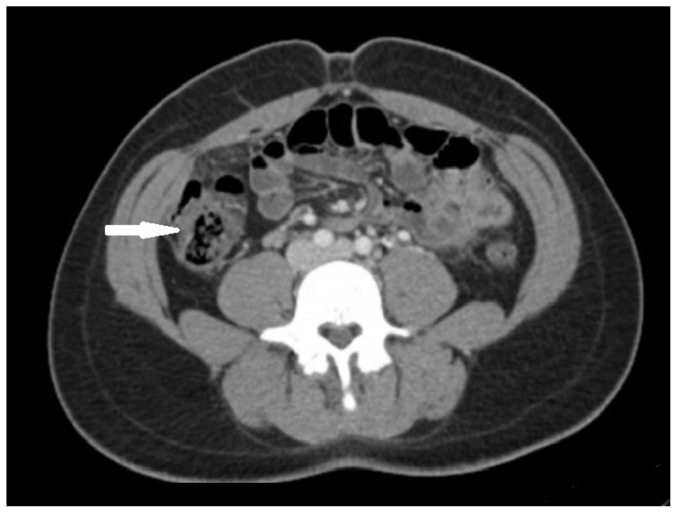

## Introduction

Fitz-Hugh Curtis syndrome (FHCS) is an uncommon sequela of sexually transmitted infections (STI).^1^,^2^ FHCS involves the spread of infection to the capsule of the liver, causing right upper quadrant or flank pain, which results in a broad list of potential differential diagnoses.^1^ The gold standard for diagnosing FHCS is the classic “violin string adhesions” on the liver that is identified during diagnostic laparoscopy.^2^ However, the diagnosis may also be suggested by the clinical picture and imaging, with laparoscopy typically reserved for patients who fail antibiotic therapy.^3^ Fitz-Hugh Curtis syndrome was once thought to be the result of either *Chlamydia trachomatis* or *Neisseria gonorrhea*, but it is now known to be polymicrobial.^3^ Therefore, an antibiotic regimen that provides anaerobic coverage is now recommended.^4^

## Presenting concerns and clinical findings

A gravida one, para one (G1P1) 25-year-old female with a history of *Chlamydia* infection presented to the emergency department (ED) with two weeks of thick, green, malodorous vaginal discharge, and associated chills and abdominal pain that began 12 hours prior to arrival. The patient denied associated nausea, vomiting, or documented fevers. She reported testing negative for STI several weeks ago at a routine well-woman visit. Her past medical history includes asthma, diet-controlled diabetes mellitus, and iron deficiency due to heavy menstrual periods. Surgical history includes cesarean section two years ago. She has been sexually active with one male partner. In regards to social history, she reported smoking 2–3 cigarettes per day and occasional alcohol intake but denied other substance use.

On examination, she was tender to palpation in the right upper quadrant, epigastrium, and left lower quadrant. She had hepatomegaly extending three centimeters below the costal margin, and Murphy’s sign was positive. Exam was negative for rebound, guarding, and costovertebral angle tenderness. Urinalysis demonstrated elevated leukocyte esterase, and her urine pregnancy test was negative. Complete metabolic panel showed normal renal function and liver function tests. Complete blood count with differential showed mild leukocytosis (white blood cell count: 16.0 x 10^3^ cells/mm^3^) and decreased hemoglobin/hematocrit (hemoglobin: 8.8 g/dL; hematocrit: 28.8%), and morphology demonstrated anisocytosis, microcytes, and hypochromia. The patient tested negative for *Chlamydia trachomatis* and *Neisseria gonorrhea*. Wet mount was positive for leukocytes, but negative for *Trichomonas vaginalis*, clue cells, or yeast. Differential diagnoses considered included cholecystitis, hepatitis, pyelonephritis, and complications of STI such as pelvic inflammatory disease (PID) or FHCS.

## Significant findings

A sagittal view from computed tomography (CT) of the abdomen and pelvis demonstrated fat stranding beneath the inferior margin of the liver (outlined in red). The axial view showed fat stranding adjacent to the ascending colon without significant colon wall thickening (arrow). Fat stranding can occur as a hazy increased attenuation (brightness) or a more distinct reticular pattern.^5^

## Patient course

Given the clinical scenario and CT findings, the patient was diagnosed with FHCS. She received intravenous (IV) fluids and 48 hours of IV cefoxitin and metronidazole as well as oral doxycycline. After 48 hours of parenteral antibiotics, her pain was controlled and she was able to tolerate oral antibiotics. Polymerase chain reaction testing for *Chlamydia* and *N. gonorrhea* was negative. She was discharged with a 14-day course of oral doxycycline and metronidazole.

## Discussion

Fitz-Hugh-Curtis syndrome, or perihepatitis, is defined as an inflammation of the liver capsule that does not involve the liver parenchyma.^1^ It occurs in 4%–27% of cases of PID.^1^,^2^ PID is a polymicrobial infection of the upper genital tract due to *Chlamydia trachomatis*, *Neisseria gonorrheae*, *Mycoplasma genitalium*, and anaerobes such as *Peptostreptococcus spp.* and *Prevotella spp*.^3^ Right upper quadrant pain in a young woman with risk factors or symptoms suggestive of STI should raise suspicion for FHCS.^1^,^2^ The differential diagnosis for FHCS is broad and includes cholecystitis, biliary colic, appendicitis, liver abscess, duodenal ulcer, and pyelonephritis.^1^ Imaging is often required to exclude other diagnoses; however, it is also possible to identify findings that suggest FHCS as a diagnosis. Abdominal and pelvis CT may show enhancement of the liver capsule, mesenteric fat stranding, small-volume ascites, or tubo-ovarian abscess.^6^ The classic finding of FHCS is the presence of “violin string adhesions” or peritoneal adhesions, between the diaphragm and the anterior surface of the liver capsule visualized during laparoscopic exploration.^2^, ^7^

One limitation of this case is the lack of confirmatory laparoscopy to support the patient’s diagnosis; however, the diagnosis was sufficiently supported by the patient’s clinical symptoms and pattern of inflammation on CT. Abdominal and pelvis CT, rather than invasive laparoscopy, is now the main diagnostic tool for FHCS; however, the sensitivity and specificity of CT have not yet been definitively determined.^6^ Wang’s retrospective study of seventeen patients with FHCS offers some evidence of the conclusiveness of CT findings in determining a diagnosis of FHCS: all seventeen patients in the study demonstrated liver inflammation on CT, with or without normal liver enzymes.^6^ If abdominal and pelvis CT findings are unremarkable yet clinical suspicion for FHCS remains high, it may be worthwhile to consult obstetrics and gynecology.

Like PID, FHCS is primarily a clinical diagnosis where imaging is utilized to rule out other causes. Determination of preexisting PID is helpful in establishing a diagnosis of FHCS. The Centers for Disease Control and Prevention (CDC) has set several criteria to aid in the clinical diagnosis of PID.^3^ Minimal criteria for PID diagnosis are determined to have high sensitivity and low specificity and include cervical motion tenderness, adnexal tenderness, or uterine tenderness.^3^ At least one minimum criterion must be present for diagnosis.^3^ The CDC has also established criteria which support but are not necessary for the diagnosis of PID.^3^ These include fever, elevated erythrocyte sedimentation rate, elevated c-reactive protein, abnormal vaginal discharge, leukocytosis, white blood cells on wet mount, and laboratory evidence of *N. gonorrhoeae* or *C. trachomatis* infection.^3^

The patient’s positive response to antibiotic treatment also played a role in confirming her diagnosis. Current guidelines for antibiotic treatment of PID and FHCS stress coverage of *N. gonorrhoeae*, *C. trachomatis*, as well as anaerobes, due to increasing evidence of anaerobic involvement in the pathogenesis of PID and FHCS.^3^,^4^ The CDC urges physicians to employ anaerobic coverage until regimens that do not cover anaerobes are shown to be effective in the prevention of long-term sequelae.^4^ Additional consideration is merited in the face of increasing antibiotic resistance, and local resistance patterns may require a change in the usual regimens.^3^ Guidelines have been changed as recently as the past decade to discourage the use of fluoroquinolones and cefixime due to the growing resistance of *N. gonorrhoeae* to these antibiotics.^3^ The CDC recommends three possible parenteral antibiotic regimens for treatment: a cephalosporin (cefotetan or cefoxitin) plus doxycycline, clindamycin plus gentamicin, or ampicillin/sulbactam plus doxycycline.^4^ In cases complicated by a tubo-ovarian abscess, clindamycin and metronidazole should be given to provide additional anaerobic coverage.^4^ For milder cases of PID, the CDC recommends a cephalosporin (ceftriaxone or cefoxitin) given intramuscularly plus oral doxycycline with or without oral metronidazole.^4^ Long-term sequalae of untreated PID/FHCS include tubal infertility, ectopic pregnancy, bowel obstruction due to adhesions, and chronic pelvic pain.^2^,^3^ In summary, FHCS is a clinical diagnosis that is supported by evidence of PID and signs of liver involvement.^3^ CT is generally utilized to rule out other possible causes but can also demonstrate signs suggesting FHCS, such as fat stranding.^4^ Liver adhesions typically resolve following antibiotic treatment targeted at the causative microbes; however, laparoscopic adhesiolysis may be required in cases refractory to antibiotic therapy.^2^ A diagnosis of FHCS requires careful consideration of antibiotic treatment options because guidelines are constantly changing to accommodate rising antibiotic resistance. Current guidelines support the use of a cephalosporin plus doxycycline and additional anaerobic coverage with metronidazole.

## Supplementary Information










