# Supplementary figures and images for: Fitz Hugh Curtis Case Report
Source: J Educ Teach Emerg Med. 2020 Apr 15;5(2):V19–21. doi: 10.21980/J82K9G (PMC10332567; doi:10.21980/J82K9G)

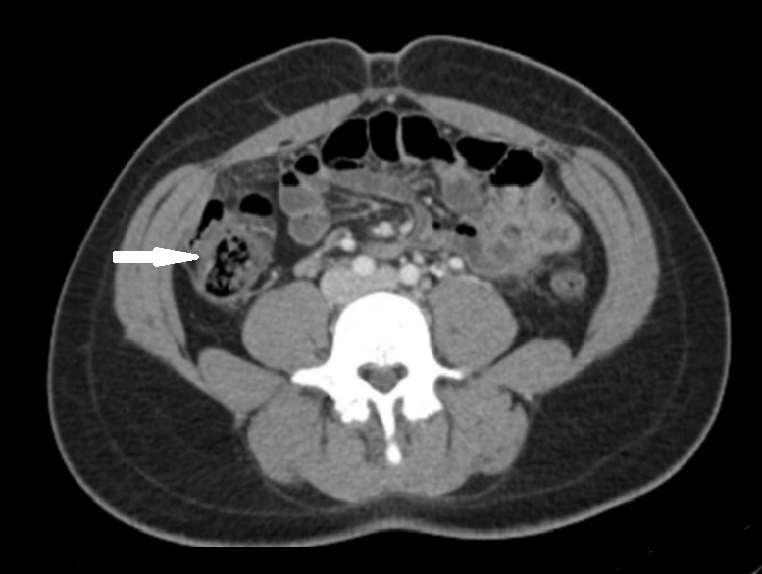

Supplement: Supplementary file 1 [file jetem-5-2-v19-supp1.jpg]

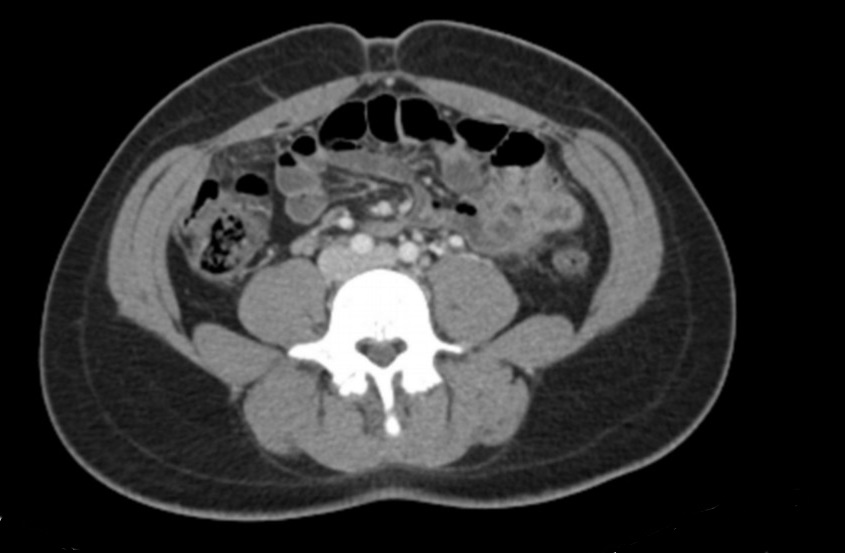

Supplement: Supplementary file 2 [file jetem-5-2-v19-supp2.jpg]

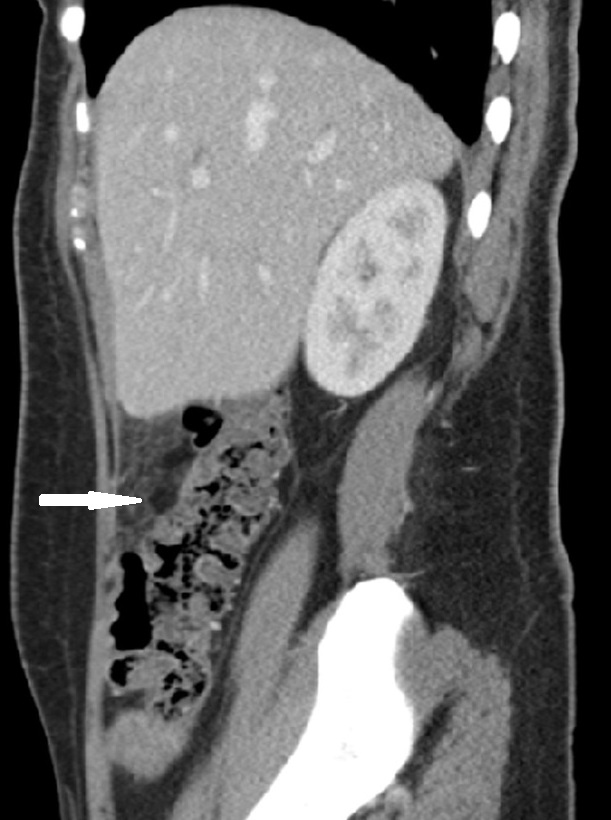

Supplement: Supplementary file 3 [file jetem-5-2-v19-supp3.jpg]

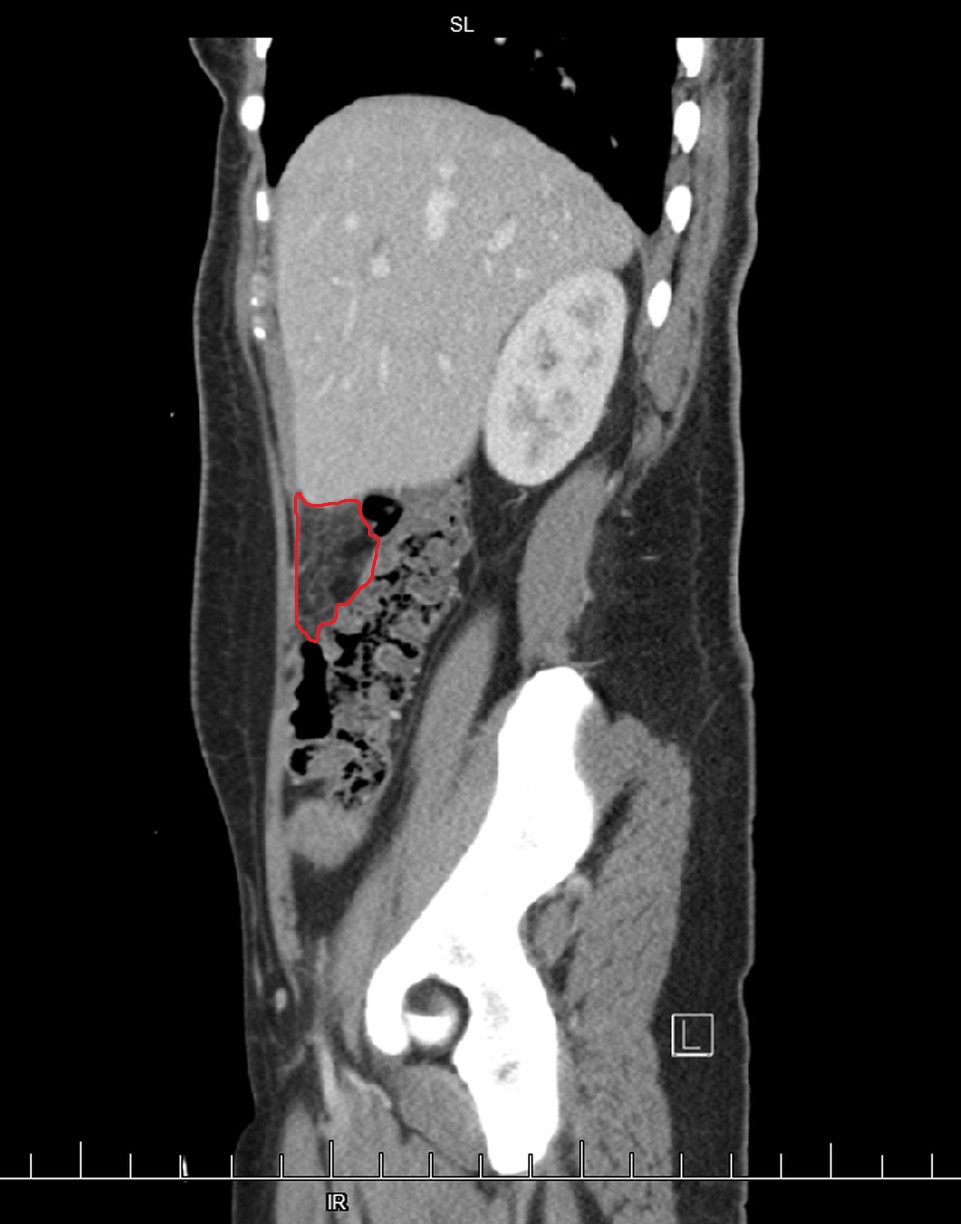

Supplement: Supplementary file 4 [file jetem-5-2-v19-supp4.jpg]

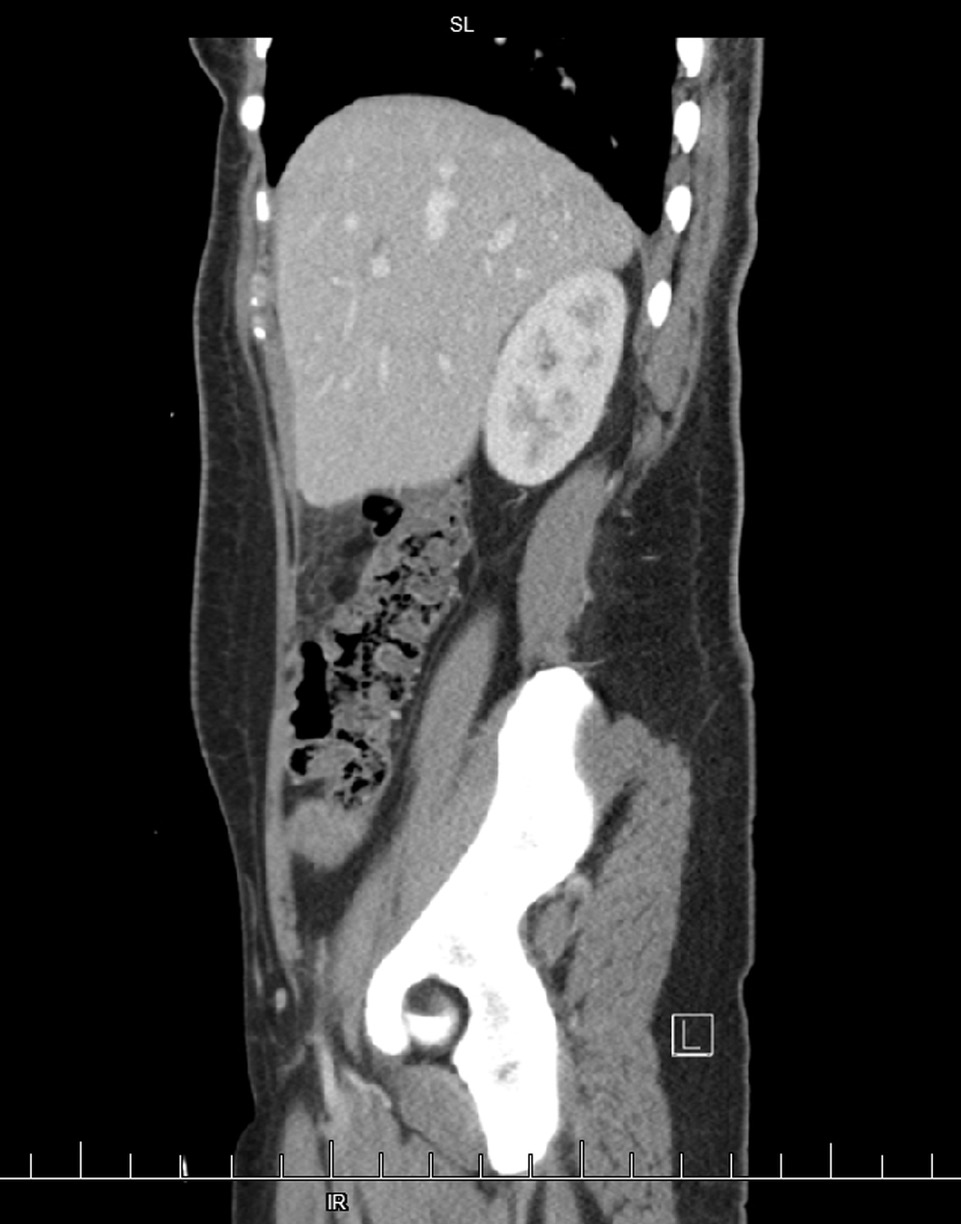

Supplement: Supplementary file 5 [file jetem-5-2-v19-supp5.jpg]
